# Economic development, alcohol consumption and life expectancy in low- and lower-middle-income countries in the Western Pacific Region: a structural equation modelling study

**DOI:** 10.1136/bmjph-2024-001453

**Published:** 2025-02-06

**Authors:** Jürgen Rehm, Huan Jiang, Ahmed S Hassan, Pol Rovira, Kevin D Shield

**Affiliations:** 1 Centre for Addiction and Mental Health, Toronto, Ontario, Canada; 2 University of Toronto, Toronto, Ontario, Canada; 3 Public Health Agency of Catalonia, Barcelona, Catalunya, Spain; 4 University Medical Center Hamburg-Eppendorf, Hamburg, Germany

**Keywords:** Public Health, Epidemiology, Health Transition, economics

## Abstract

**Introduction:**

Economic transition has historically been shown to be associated with longer life expectancy in current high-income countries. We examined the role of alcohol consumption in this transition process for lower- and middle-income countries.

**Methods:**

We tested three hypotheses on the interrelationship between economic growth, level of alcohol consumption and life expectancy in all six countries in the WHO Western Pacific Region, which transitioned from low- to lower-middle-income countries over the past 20 years. Structural equation modelling, corrected for autoregressive effects, was used to test the association between economic development and life expectancy, adult per capita consumption of alcohol, the prevalence of past-year drinkers and alcohol-attributable mortality. The direct impact of alcohol per capita consumption (APC) on life expectancy was also estimated.

**Results:**

Overall, economic development was strongly positively associated with both life expectancy and alcohol consumption, and a higher level of alcohol consumption resulted in a lowered life expectancy, when directly measured. Thus, changes in gross domestic product per capita at purchasing power parity of $ 1000 Int. were linked to changes in the same direction in life expectancy of 0.94% (95% CI 0.66%, 1.21%) and with an increase in APC of 76.8% (55.38%, 98.3%). Average loss in life expectancy due to alcohol consumption was 1.76 (0.81, 2.72) years for males and 0.59 (0.12, 1.07) for females. There was heterogeneity found between countries.

**Conclusion:**

Alcohol consumption is expected to increase in an economic transition from a low- to lower-middle-income country and to have a negative impact on life expectancy. Alcohol control policies should be enacted to reap the full health benefits of economic growth.

**WHAT IS ALREADY KNOWN ON THIS TOPIC**

- Angus Deaton in *The Great Escape: Health, Wealth, and the Origins of Inequality* developed a conceptual framework on economy and health, which was expanded to include alcohol consumption. Overall, there are few studies on the topic of economic development, alcohol consumption and life expectancy in low- and lower-middle-income countries.

**WHAT THIS STUDY ADDS**

- This study is the first systematic test of the conceptual framework on the role of alcohol consumption in the economic transition of countries, using data from the six countries in the WHO Western Pacific Region that transitioned from low- to lower-middle-income countries over the past 20 years: Cambodia (transition from low- to lower-middle-income country: 2015); Lao People’s Democratic Republic (2010); Mongolia (2007); Papua New Guinea (2008); Solomon Islands (2010); and Vietnam (2009). Economic development was strongly associated with both life expectancy and alcohol consumption, and a higher level of alcohol consumption resulted in a lowered life expectancy when directly measured. There was some heterogeneity between countries, which may in part be related to the implementation of alcohol control policies.

**HOW THIS STUDY MIGHT AFFECT RESEARCH, PRACTICE OR POLICY**

- Alcohol consumption is expected to increase during economic transitions of countries moving from low- to lower-middle income and can act as barrier to a country reaping the full health benefits of its economic growth. Strong alcohol control policies at the population level can decrease the level of alcohol consumption and increase life expectancy. More research in these mechanisms is needed.

## Introduction

**Figure 1:**
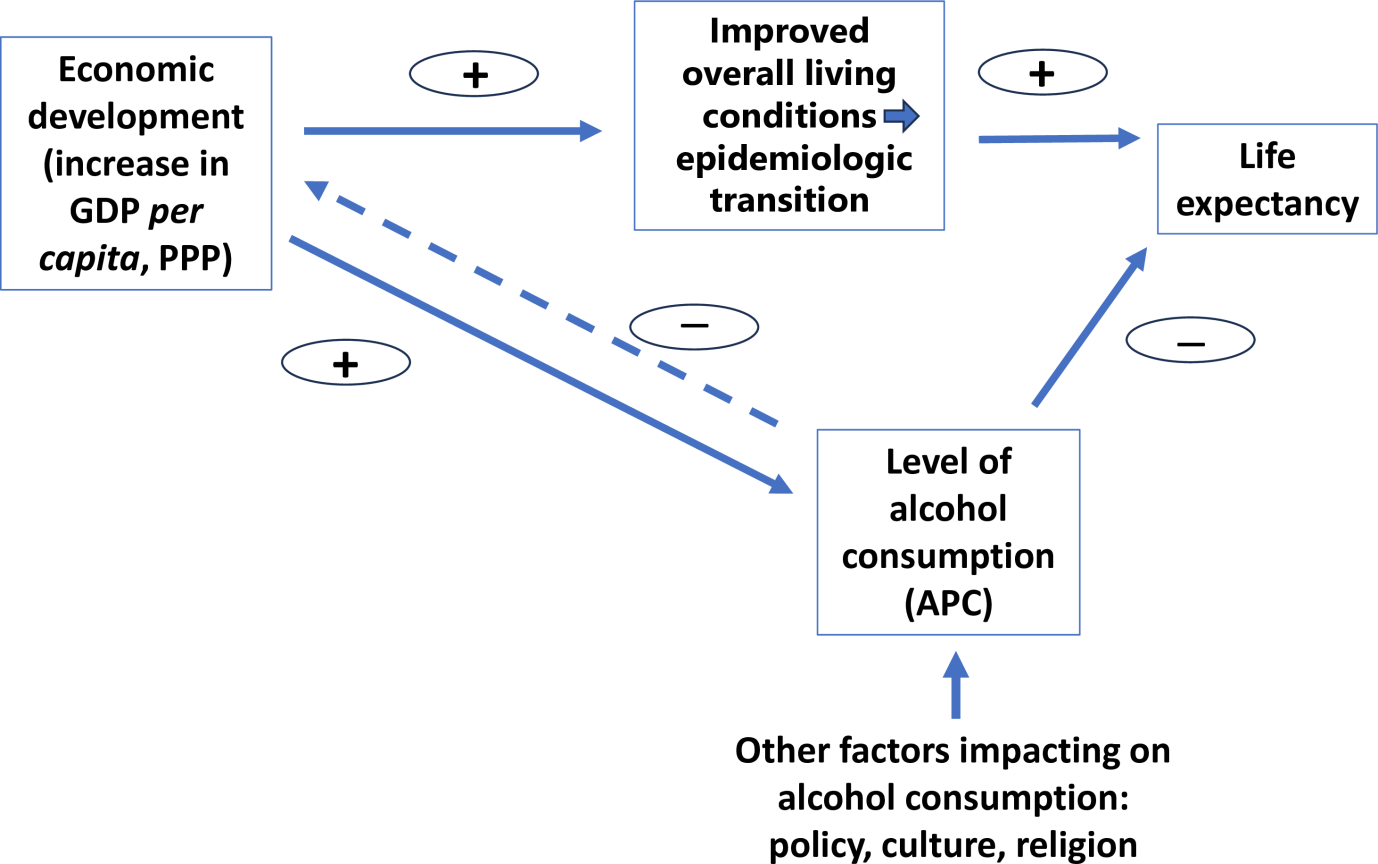
Conceptual model of economic development, alcohol consumption and life expectancy. APC, alcohol per capita consumption; GDP, gross domestic product; PPP, purchasing power parity.

 In his book *The Great Escape: Health, Wealth, and the Origins of Inequality*, Angus Deaton sketched out an historical trajectory spanning two centuries on the health and wealth of current high-income countries. His analyses show that economic development not only led to wealthier and much healthier populations but also created higher inequalities within societies, as well as between societies globally.^1^ This framework has been recently extended to include alcohol consumption and its role in economic development and progress in health.^2^ The core of the resulting conceptual model including alcohol use is illustrated in figure 1.

The upper trajectory of this figure shows how economic development impacts life expectancy as a summary indicator of health^3^ via the improved living conditions facilitating an epidemiologic transition (ie, the main findings of Deaton).^1^ It should be noted that part of this transition, particularly the decreases in infectious diseases, could also be achieved through the use of medical knowledge, technology and affordable medication obtained from the high-income countries.

The lower trajectory indicates the positive impact of economic development on the level of alcohol consumption, as measured in adult (defined as 15 years old and older) alcohol per capita consumption (APC), the best indicator for population level of drinking.^4 5^ APC is also one of the indicators for the Sustainable Development Goals (3.5.2^6 7^). The second part of the lower trajectory highlights the potential negative impact of increased APC on life expectancy.

The marked positive association between economic development and alcohol consumption for low- and lower-middle-income countries has been established in several papers: the wealthier a country, the higher the availability and affordability of alcohol,^8^ in part due to higher disposable income for its inhabitants, leading to overall levels of consumption.^9^,^12^ However, most of these associations reported in the literature are cross-sectional, and, to our knowledge, there has been no detailed longitudinal investigation comparing different countries, or other attempts to establish the causality of these relations.

In addition, there is a negative impact of level of consumption on life expectancy mediated by alcohol-attributable diseases and deaths (for an overview, see^13^), which would not have happened in absence of alcohol consumption.^6^ Alcohol consumption has been established as a key risk factor in all comparative risk assessments to date^14^ (for an early WHO report, see^15^).

There is an additional arrow in figure 1 concerning APC which requires an explanation: the dotted arrow shows that APC has a negative impact on a country’s economic transition. In their last models on the impact of alcohol consumption, the Organisation for Economic Co-operation and Development (OECD) found that, on a macroeconomic level, exceeding alcohol consumption above the 1/1.5 drinks per day cap for females/males leads to a 1.6% lower gross domestic product (GDP) due to alcohol-attributable diseases.^16^ However, the OECD membership rules stipulate that countries must be significant players in the global economy, and this effectively excludes low and lower-middle income countries. A recent review and meta-analysis that included such countries—which has yet to be peer-reviewed—found a negative effect from the level of alcohol consumption on GDP.^17^ Moreover, there are some more qualitative discussions regarding the potential existence of a bidirectional arrow between economic development and alcohol consumption in earlier reviews.^18 19^ This arrow is dotted as the stronger impact seems to flow directly from economic development to APC but only indirectly in the opposite direction, and because we will not attempt to estimate its value in this article.

This publication will attempt to empirically test key assumptions of the conceptual framework in particular: (a) the postulated positive impact of economic development on life expectancy and APC and (b) the postulated negative impact of APC on life expectancy. For the empirical test of the conceptual model, we selected the WHO region with the highest gradient of economic development in the past 20 years as a test case, with many countries transitioning from low- to lower-middle-income countries (as defined by the World Bank).^20^ We identified these transitioning countries and included them in the empirical study if they had not reverted back within 5 years: Cambodia (transition from low- to lower-middle-income country: 2015); Lao People’s Democratic Republic (Lao PDR; 2010); Mongolia (2007); Papua New Guinea (2008); Solomon Islands (2010); and Vietnam (2009).

## Methods

### Data

For all selected countries, we collected data from the World Bank for 10 years prior to and 10 years following the transition on GDP per capita at purchasing power parity (GDP PPP)^21^ and on life expectancy (total and separately by sex) and from the WHO Global Health Observatory on life tables.^22 23^ Data for APC, current drinking and alcohol-attributable mortality were obtained from the WHO’s ‘Global Information System on Alcohol and Health’.^12 24^ Since Cambodia transitioned in 2015, only 6 years of data following the transition were available. All data on these indicators can be found in online supplemental material S1.

### Statistical methods

To describe the data, we used annual growth rates and Pearson correlation, separated into the 10-year periods prior and following the transition from low- to lower-middle-income, and separated by sex, where applicable.

Structural equation modelling (SEM)^25^ was used to estimate the complex relationships between APC, GDP PPP and life expectancy. Their relationships were determined through path analysis, where path coefficients were estimated to quantify the importance of presumed causative factors on outcomes and were computed simultaneously for all endogenous variables. As figure 1 indicates, two structural equations were incorporated: the first one pertained to life expectancy and its associations with APC and GDP PPP, while the second one focused on APC and its association with GDP PPP, with both equations allowing for nested random effects among countries and temporal autocorrelation of observations. For better interpretability of results, the outcomes were log-transformed, and GDP PPP was entered in units of $ 1000 Int. Potential temporal autocorrelation of observations was modelled by using a continuous autoregressive structure in both equations. They were further tested by comparing Akaike’s information criterion (AIC)—the model-fitting statistics—with the results, without taking into account any temporal autocorrelations.^26^ In addition, cross-correlations were examined to identify any possible lag structures for APC and GDP PPP; these analyses do not incorporate any lag structure.

As a sensitivity analysis, a set of structure equations additionally including calendar year as an independent predictor were analysed to test the stability of the system of interactions. The statistical software R (V.4.0.2),^27^ and the R package PIECEWISESEM was used to perform all path analyses.^28^ The underlying formula and the cross-correlation structure can be seen in online supplemental material S2.

In addition, the effect of APC on life expectancy was directly estimated using abridged life tables.^22^ Concretely, changes in life expectancy due to alcohol consumption were calculated using these life tables alongside alcohol-attributable mortality statistics from the WHO, for the country-specific transition year or the closest year available from the WHO’s global status reports. Sex- and country-specific alterations in life expectancy under the counterfactual scenario where no alcohol-attributable deaths occurred were derived using abridged life tables. Specifically, values from life tables representing the probability of mortality between ages x and x+n (referred to as nqx) were modified by multiplying the mortality probability by the respective fraction of deaths not attributable to alcohol consumption for the corresponding age bracket.

## Findings

### Descriptive results

Table 1 gives an overview of the growth rates of the main indicators relevant for the framework. The GDP PPP grew steadily in all six countries over the 21 points of observation (10 years prior and 10 years following the transition plus the transition year; the exception was Cambodia with 17 years of observation), on average $ 4200 Int. (95% CI 3746, 4654). All the other indicators had proportionally slower growth in the 10 years after transition and more between-country variability. Most drastically, the annual APC growth was 5.92% in the 10 years before the transition but amounted to 0.34% in the 10 years after, although with high variability. The prevalence of past year alcohol consumption also increased more before than after the transition, again with high variability. It should be noted that for Cambodia, Lao PDR and the Solomon Islands, the 10 years following the transition to a lower-middle-income status included at least 1 year affected by COVID-19, which was associated with decreases in all indicators (for GDP PPP per capita, see ^21^ for life expectancy and ^23^ for the alcohol indicators).^12^

**Table 1:** Annual growth rates for key indicators of economic development, alcohol consumption and health 10 years prior to and following the transition from a low- to lower-middle-income country

Country (year of transition from low- to lower-middle income)	GDP PPP		Life expectancy at birth		APC in litres ethanol		Prevalence of past year drinkers	
	Before	After	Before	After	Before	After	Before	After
Cambodia (2015)	7.04%	5.87%	0.84%	−0.07%	9.04%	−0.86%	2.24%	−0.19%
Lao PDR (2010)	7.73%	8.06%	0.92%	0.69%	0.78%	0.08%	0.29%	0.33%
Mongolia (2007)	7.23%	5.54%	0.82%	0.63%	12.85%	−2.67%	4.06%	−0.22%
Papua New Guinea (2008)	1.24%	4.30%	0.32%	0.41%	−2.18%	−0.89%	−0.12%	0.00%
Solomon Islands (2010)	3.41%	1.94%	0.22%	0.21%	0.60%	3.10%	0.10%	0.32%
Vietnam (2009)	7.91%	7.76%	0.16%	0.08%	14.40%	3.27%	2.15%	1.05%
Average growth rate	5.76%	5.58%	0.55%	0.33%	5.92%	0.34%	1.45%	0.22%
(95% CI)	(2.86%, 8.66%)	(3.19%, 7.97%)	(0.18%, 0.91%)	(0.01%, 0.64%)	(−1.50%, 13.34%)	(−2.16%, 2.84%)	(−0.28%, 3.18%)	(−0.28%, 0.71%)

Cambodia: data were only available for 6 years for GDP PPP, life expectancy (until 2021) and APC after the transition from low- to lower-middle-income in 2015.

Prevalence of past year drinkers: available data before status change: Mongolia (seven periods), Papua New Guinea (eight periods) and Vietnam (nine periods). Available data after status change: Cambodia (five periods).

APC, alcohol per capita consumption; GDP PPP, Gross domestic product per capita at purchasing power parity; LAO PDR, Lao People’s Democratic Republic.

The correlational structure of the two direct pathways from GDP PPP to life expectancy and to APC showed high correlations, both overall and before and after the transition: average Pearson correlation (95% CI) between GDP PPP and life expectancy (0.63 (0.001, 1.00) (before, 0.52 (−0.10, 1.00); after, 0.56 (0.19, 0.93))) and average Pearson correlation between GDP PPP and APC (0.62 (0.05, 1.00) (before, 0.64 (0.15, 1.00); after, 0.53 (0.23, 0.82))). The country-specific correlations and their 95% CIs can be found in online supplemental material S3.

While these descriptive results corroborate the conceptual model, they are based on correlations of time-series data which are prone to bias of spurious correlation.

### Testing the conceptual model

Table 2 gives an overview of the key results of the SEM model. An increase in $ 1000 Int. was found to be associated with an increase of 0.94% (0.66%, 1.21%) in life expectancy. While the anticipated negative association between APC and life expectancy was observed, the effect size was small, and the association was not statistically significant. As predicted, GDP PPP exhibited a positive correlation with APC; specifically, an increase in $ 1000 Int. was related to a 76.80% (55.28%, 98.32%) increase in APC. The SEM model was an adequate fit to the data based on output from a χ^2^ goodness-of-fit test and the AIC statistics (p=0.37, AIC=−541.91).

**Table 2:** Impact of economic development and level of alcohol consumption on life expectancy

Outcome	Predictor	Estimate	SE	df	P Value
Life expectancy	GDP PPP	0.0094	0.0014	114	<0.0001
Life expectancy	APC	−0.0004	0.0005	114	0.4731
APC	GDP PPP	0.7680	0.1098	115	<0.0001

APC, alcohol per capita consumption in litres ethanol; GDP PPP, gross domestic product per capita in $ 1000 Int., at Purchasing Power Parity.

The sensitivity model confirmed the main relationships, with relatively similar adjusted values for life expectancy between countries at the transition from low- to lower-middle income country (between 63.4 in Lao PDR and 73.0 in Vietnam), but the large country differences between APC at the same time point remain after adjustment for GPP PPP (between 2.9 for Solomon Islands and 9.6 for Lao PDR; online supplemental material S4; for unadjusted values see online supplemental material S1).

Results of the direct estimation of the loss of life expectancy indicate that, on average, life expectancy would have been 1.76 (0.81, 2.72) years higher for males and 0.59 (0.12, 1.07) years higher for females without the consumption of alcohol. The highest differences for both sexes were found in Mongolia (males, 3.03 years; females, 1.38 years) and the lowest in the Solomon Islands (males, 0.72 years; females, 0.21 years). More details on the direct estimation can be found in online supplemental material S5.

## Discussion

Overall, an examination of the conceptual model regarding the role of alcohol in economic development and health in all of the countries of the WHO Western Pacific Region that transitioned from low- to lower-middle-income status within the past 20 years corroborated the basic assumptions: economic development was strongly associated with both life expectancy and alcohol consumption, and a higher level of alcohol consumption resulted in a lowered life expectancy, when directly measured.

Before exploring the implications of these findings, we would like to point out the potential limitations of our findings. First, our sample was limited to six countries having relatively high variability. On the one hand, countries like Cambodia, Lao PDR, Vietnam and Mongolia had continuously high economic growth, resulting in increases in life expectancy and of alcohol consumption, especially in the 10 years prior to the transition. On the other hand, economic growth was less pronounced in a country like Papua New Guinea, with no associated growth in alcohol consumption and lower gains in life expectancy. Thus, other factors need to be considered to explain the variation between countries. Second, we did not have enough data to estimate the complex non-recursive relationship between economic growth and alcohol consumption, that is, the fact that there is a main effect from economic growth on alcohol consumption, but also a feedback loop. Future research is needed to examine the impact of alcohol consumption on economic growth in low- and lower-middle-income countries, similar to what the OECD did for high-income countries. Some of this research should involve a more detailed model specifying the pathway leading from APC to GDP PPP. Such specification will allow us to better disentangle the feedback loop described above. Regarding the direct estimation of alcohol-attributable losses to life expectancy, there is the assumption that these deaths would not have occurred in the same year without consumption of alcohol and that no deaths from different causes occurred in that year. This assumption is difficult to test, but statistical modelling based on abrupt changes in population alcohol consumption due to policy suggest that these underlying assumptions may be realistic.^29 30^

Finally, all analyses are only as good as the underlying data. While we used standard indicators, which are currently considered to be the best choices, such as GDP PPP, life expectancy and APC, each of these measures has limitations. Thus, the quantitative increase of the economy as measured in GDP per capita often does not affect an array of individual components of human well-being, including social inequality, safety, health and civil rights, in addition to not addressing factors such as quality of institutions, depletion of natural resources and economic sustainability.^31^

Since life expectancy is only an indirect measure for health based on mortality, and does not include morbidity and disability, it has been replaced by health-adjusted life expectancy indicators in many circumstances.^32^ However, given that health adjustments are usually based on subjective data, potentially introducing bias, and must be updated regularly via standardised surveys—which is not the norm in most of the countries examined—we believe that life expectancy is the more robust comparative indicator for our analyses. Life expectancy depends only on all-cause mortality and as all countries examined have a vital registration system (for details on each system, see ^11^), all-cause mortality should be relatively accurately reported. More bias may be expected for alcohol-attributable mortality, which is based on specific causes of death that are often stigmatised (eg, liver cirrhosis).^33 34^ Thus, the direct estimates of years of life lost due to alcohol consumption may be underestimated and may have higher confidence intervals than those reported.

Finally, while alcohol is usually considered as one of the risk factors with the least measurement error,^35^ the component of unrecorded consumption, which makes up a relatively high proportion in several countries,^11 12^ may contain some measurement error. It also has the weakness that its impact depends on the prevalence of drinking; the same APC with a higher number of abstainers has more impact on health and thus APC per drinker has been considered to be the better indicator to be used within a health framework.^6^ Future research will need to use and compare multiple indicators to explore the relationships between economic development, alcohol consumption and life expectancy.

However, even at this point, we can sketch out the role of alcohol consumption in the economic transition and its impact on health. Basically, alcohol consumption seems to hinder societies reaping the full benefits of economic transition. In a positive economic transition, countries increase their GDP PPP, which impacts positively on living conditions and health. There is a parallel epidemiologic transition away from infectious disease and causes of death toward a higher proportion of chronic diseases and causes of death which happen later in life, thus increasing life expectancy.^36^ Economic transition is also often associated with an increase in APC, and for these six countries, almost 40% of the variance in APC, on average, was shared with GDP PPP. Part of the increase was driven by a higher prevalence of past year drinkers. However, as alcohol consumption also impacts burden of disease, both infectious and chronic,^37^ a portion of the positive effect of the economic transition on health is lost by alcohol consumption. Thus, an important indicator of the use of alcohol’s impact on health should be loss in life expectancy or loss of healthy life expectancy.

So far, we have described the usual processes involving alcohol consumption during an economic transition from low- to lower-middle-income status. However, there is variation between countries as evidenced in table 1 above and in the online supplemental materials (in particular S4 and S5). Other factors also have an impact on the level of alcohol consumption, such as religion,^13^ culture or alcohol control policy.^38^ One important source of variation here is alcohol control policy. Alcohol control policy is an important tool countries use to reduce the level of alcohol consumption and its impact on life expectancy.^39^ For instance, a recent investigation of major evidence-based alcohol policies (for classification, see ^38 40^)—over two decades in four high-income countries in Europe with an overall high level of alcohol consumption—showed that each such policy was related to an average reduction in consumption of 0.83 (0.41; 1.21) litres (L) of APC per year (1.21 L, 0.41 L) in the same year, with no significant differences between countries.^41^ To our knowledge, few such studies exist on the impact of alcohol control policies in low- and lower-middle-income countries (for an exception, see ^42^), but clearly we would expect decreases in APC and subsequent increases in life expectancy if strong alcohol policies affecting availability, affordability, and acceptability were to be implemented.^40^

In summary, alcohol consumption is expected to increase in an economic transition from low- to lower-middle-income countries, having a negative impact on life expectancy. Alcohol control policies should be enacted in such countries for them to reap the full health benefits of economic growth.

## Supplementary material

10.1136/bmjph-2024-001453 online supplemental file 1

## Data Availability

All data relevant to the study are included in the article or uploaded as supplementary information.
