# Supplementary material for: Economic development, alcohol consumption and life expectancy in low- and lower-middle-income countries in the Western Pacific Region: a structural equation modelling study
Source: BMJ Public Health. 2025 Feb 6;3(1):e001453. doi: 10.1136/bmjph-2024-001453 (PMC11816530; doi:10.1136/bmjph-2024-001453)
Supplement: online supplemental file 1 [file bmjph-3-1-s001.docx]

# Supplementary materials Economic development, alcohol consumption and life expectancy in low- and lower-middle-income countries in the Western Pacific Region – a structural equation modelling study

**Table of Contents**

[Supplementary materials Economic development, alcohol consumption and life expectancy in low- and lower-middle-income countries in the Western Pacific Region – a structural equation modelling study 1](#_Toc188088152)

[S1: Key indicators of economic development, alcohol use, and health 10 years prior to and following the transition from a low- to lower-middle-income country 2](#_Toc188088153)

[S2: Specification of SEM model, sensitivity analysis and cross-correlation structure 5](#_Toc188088154)

[Specification for main analysis 5](#_Toc188088155)

[Specification for sensitivity analysis 5](#_Toc188088156)

[Cross-correlation structure: no lag was identified 6](#_Toc188088157)

[S3: Country-specific correlations and their 95% confidence intervals 7](#_Toc188088158)

[**Table 3.1:** Correlation analysis of adult alcohol per capita (in litres) consumption and life expectancy for overall data as well as before and after the transition from low- to lower-middle income 7](#_Toc188088159)

[**Table 3.2**: Correlation analysis of GDP PPP and adult alcohol per capita (in litres) consumption for overall data as well as prior to and following the transition from low- to lower-middle income 8](#_Toc188088160)

[S4: Sensitivity analysis of the SEM model: relationship between economic development, level of alcohol consumption, and life expectancy (GDP PPP in units of $ 1000 Int.) 9](#_Toc188088161)

[S5: Estimated loss of life expectancy due to alcohol consumption (direct estimation) 10](#_Toc188088162)

## S1: Key indicators of economic development, alcohol use, and health 10 years prior to and following the transition from a low- to lower-middle-income country

| **Year** | **Relative year** | **Country** | **GDP PPP** | **Life Expectancy** | **APC** | **Prevalence** |
| --- | --- | --- | --- | --- | --- | --- |
| 2005 | -10 | Cambodia | 1727.64 | 64.29 | 2.93 | 0.40 |
| 2006 | -9 | Cambodia | 1938.93 | 65.06 | 3.07 | 0.40 |
| 2007 | -8 | Cambodia | 2156.77 | 65.73 | 3.32 | 0.41 |
| 2008 | -7 | Cambodia | 2306.69 | 66.47 | 3.46 | 0.42 |
| 2009 | -6 | Cambodia | 2288.72 | 67.44 | 3.60 | 0.42 |
| 2010 | -5 | Cambodia | 2418.84 | 67.71 | 5.05 | 0.45 |
| 2011 | -4 | Cambodia | 2605.49 | 68.42 | 5.48 | 0.46 |
| 2012 | -3 | Cambodia | 2867.54 | 68.92 | 5.68 | 0.47 |
| 2013 | -2 | Cambodia | 3051.00 | 69.30 | 6.15 | 0.48 |
| 2014 | -1 | Cambodia | 3198.67 | 69.74 | 6.55 | 0.49 |
| **2015** | **0** | **Cambodia** | **3411.58** | **69.87** | **6.96** | **0.50** |
| 2016 | 1 | Cambodia | 3708.37 | 70.22 | 7.67 | 0.51 |
| 2017 | 2 | Cambodia | 3972.72 | 70.52 | 8.19 | 0.52 |
| 2018 | 3 | Cambodia | 4319.02 | 70.56 | 8.53 | 0.52 |
| 2019 | 4 | Cambodia | 4653.63 | 70.69 | 8.72 | 0.53 |
| 2020 | 5 | Cambodia | 4515.71 | 70.42 | 6.67 | 0.49 |
| 2021 | 6 | Cambodia | 4805.08 | 69.58 | 8.30 | - |
|  |  |  |  |  |  |  |
| 2000 | -10 | Lao PDR | 1793.78 | 58.38 | 9.93 | 0.54 |
| 2001 | -9 | Lao PDR | 1908.46 | 59.07 | 7.14 | 0.50 |
| 2002 | -8 | Lao PDR | 2021.29 | 59.53 | 7.46 | 0.50 |
| 2003 | -7 | Lao PDR | 2154.35 | 60.03 | 7.55 | 0.50 |
| 2004 | -6 | Lao PDR | 2320.56 | 60.29 | 7.69 | 0.51 |
| 2005 | -5 | Lao PDR | 2526.30 | 60.98 | 7.89 | 0.51 |
| 2006 | -4 | Lao PDR | 2784.18 | 61.49 | 7.89 | 0.51 |
| 2007 | -3 | Lao PDR | 3028.39 | 62.08 | 9.56 | 0.54 |
| 2008 | -2 | Lao PDR | 3276.73 | 62.60 | 9.68 | 0.54 |
| 2009 | -1 | Lao PDR | 3491.59 | 63.27 | 9.78 | 0.54 |
| **2010** | **0** | **Lao PDR** | **3778.15** | **63.97** | **10.73** | **0.56** |
| 2011 | 1 | Lao PDR | 4106.34 | 64.74 | 10.74 | 0.56 |
| 2012 | 2 | Lao PDR | 4793.83 | 65.36 | 10.65 | 0.56 |
| 2013 | 3 | Lao PDR | 5241.85 | 65.72 | 10.81 | 0.56 |
| 2014 | 4 | Lao PDR | 5828.38 | 66.36 | 11.02 | 0.57 |
| 2015 | 5 | Lao PDR | 6126.44 | 66.67 | 11.22 | 0.57 |
| 2016 | 6 | Lao PDR | 6798.36 | 67.17 | 11.69 | 0.58 |
| 2017 | 7 | Lao PDR | 7211.26 | 67.43 | 11.37 | 0.58 |
| 2018 | 8 | Lao PDR | 7727.75 | 67.63 | 11.02 | 0.57 |
| 2019 | 9 | Lao PDR | 8172.58 | 68.14 | 12.17 | 0.59 |
| 2020 | 10 | Lao PDR | 8198.84 | 68.50 | 10.82 | 0.57 |
|  |  |  |  |  |  |  |
| 1997 | -10 | Mongolia | 3318.58 | 60.82 | 2.95 | - |
| 1998 | -9 | Mongolia | 3434.32 | 61.77 | 3.33 | - |
| 1999 | -8 | Mongolia | 3555.14 | 62.53 | 3.50 | - |
| 2000 | -7 | Mongolia | 3643.61 | 62.88 | 2.72 | 0.43 |
| 2001 | -6 | Mongolia | 3802.17 | 63.28 | 2.76 | 0.43 |
| 2002 | -5 | Mongolia | 4008.49 | 63.52 | 3.02 | 0.44 |
| 2003 | -4 | Mongolia | 4335.97 | 64.21 | 2.14 | 0.42 |
| 2004 | -3 | Mongolia | 4883.73 | 64.80 | 2.17 | 0.43 |
| 2005 | -2 | Mongolia | 5357.26 | 64.88 | 5.98 | 0.50 |
| 2006 | -1 | Mongolia | 5944.02 | 65.51 | 5.37 | 0.50 |
| **2007** | **0** | **Mongolia** | **6667.24** | **65.97** | **9.86** | **0.57** |
| 2008 | 1 | Mongolia | 7320.55 | 66.38 | 9.66 | 0.57 |
| 2009 | 2 | Mongolia | 7184.47 | 66.77 | 7.35 | 0.54 |
| 2010 | 3 | Mongolia | 7631.15 | 67.18 | 8.50 | 0.56 |
| 2011 | 4 | Mongolia | 8998.69 | 67.44 | 7.42 | 0.55 |
| 2012 | 5 | Mongolia | 10346.02 | 68.11 | 8.28 | 0.56 |
| 2013 | 6 | Mongolia | 10685.72 | 68.59 | 13.15 | 0.62 |
| 2014 | 7 | Mongolia | 11198.32 | 69.05 | 10.55 | 0.59 |
| 2015 | 8 | Mongolia | 10769.25 | 69.50 | 9.08 | 0.58 |
| 2016 | 9 | Mongolia | 10832.48 | 69.87 | 9.34 | 0.58 |
| 2017 | 10 | Mongolia | 11431.50 | 70.24 | 7.53 | 0.56 |
|  |  |  |  |  |  |  |
| 1998 | -10 | Papua New Guinea | 2297.03 | 60.63 | 2.45 | - |
| 1999 | -9 | Papua New Guinea | 2291.23 | 61.67 | 2.31 | - |
| 2000 | -8 | Papua New Guinea | 2207.15 | 61.72 | 2.12 | 0.29 |
| 2001 | -7 | Papua New Guinea | 2178.91 | 61.77 | 2.29 | 0.29 |
| 2002 | -6 | Papua New Guinea | 2136.57 | 61.70 | 2.12 | 0.29 |
| 2003 | -5 | Papua New Guinea | 2153.39 | 61.80 | 1.87 | 0.28 |
| 2004 | -4 | Papua New Guinea | 2198.40 | 61.76 | 1.77 | 0.28 |
| 2005 | -3 | Papua New Guinea | 2334.89 | 61.80 | 2.06 | 0.29 |
| 2006 | -2 | Papua New Guinea | 2457.95 | 61.92 | 1.80 | 0.28 |
| 2007 | -1 | Papua New Guinea | 2637.96 | 62.03 | 1.93 | 0.28 |
| **2008** | **0** | **Papua New Guinea** | **2599.12** | **62.57** | **1.96** | **0.28** |
| 2009 | 1 | Papua New Guinea | 2709.81 | 62.79 | 1.88 | 0.28 |
| 2010 | 2 | Papua New Guinea | 2930.77 | 63.04 | 1.11 | 0.27 |
| 2011 | 3 | Papua New Guinea | 2938.25 | 63.53 | 1.21 | 0.27 |
| 2012 | 4 | Papua New Guinea | 3046.78 | 63.73 | 1.41 | 0.27 |
| 2013 | 5 | Papua New Guinea | 3133.19 | 63.96 | 1.46 | 0.28 |
| 2014 | 6 | Papua New Guinea | 3530.50 | 64.26 | 1.81 | 0.28 |
| 2015 | 7 | Papua New Guinea | 3704.96 | 64.70 | 1.74 | 0.28 |
| 2016 | 8 | Papua New Guinea | 3851.27 | 64.84 | 1.64 | 0.28 |
| 2017 | 9 | Papua New Guinea | 3967.02 | 65.10 | 1.85 | 0.29 |
| 2018 | 10 | Papua New Guinea | 3957.93 | 65.18 | 1.80 | 0.28 |
| 2000 | -10 | Solomon Islands | 1539.38 | 67.23 | 1.01 | 0.26 |
| 2001 | -9 | Solomon Islands | 1414.55 | 67.36 | 0.42 | 0.25 |
| 2002 | -8 | Solomon Islands | 1364.26 | 67.52 | 0.84 | 0.25 |
| 2003 | -7 | Solomon Islands | 1448.35 | 67.64 | 0.72 | 0.25 |
| 2004 | -6 | Solomon Islands | 1565.62 | 67.80 | 1.18 | 0.26 |
| 2005 | -5 | Solomon Islands | 1695.07 | 67.94 | 0.95 | 0.26 |
| 2006 | -4 | Solomon Islands | 1779.33 | 68.09 | 1.00 | 0.26 |
| 2007 | -3 | Solomon Islands | 1851.93 | 67.99 | 1.08 | 0.26 |
| 2008 | -2 | Solomon Islands | 1960.74 | 68.40 | 1.06 | 0.26 |
| 2009 | -1 | Solomon Islands | 1984.32 | 68.47 | 1.27 | 0.26 |
| **2010** | **0** | **Solomon Islands** | **2151.92** | **68.73** | **1.07** | **0.26** |
| 2011 | 1 | Solomon Islands | 2303.04 | 68.91 | 1.01 | 0.26 |
| 2012 | 2 | Solomon Islands | 2344.66 | 69.08 | 0.94 | 0.26 |
| 2013 | 3 | Solomon Islands | 2447.58 | 69.19 | 0.91 | 0.26 |
| 2014 | 4 | Solomon Islands | 2459.60 | 69.23 | 0.93 | 0.26 |
| 2015 | 5 | Solomon Islands | 2462.83 | 69.58 | 0.98 | 0.26 |
| 2016 | 6 | Solomon Islands | 2561.14 | 69.80 | 1.31 | 0.27 |
| 2017 | 7 | Solomon Islands | 2625.14 | 69.99 | 1.36 | 0.27 |
| 2018 | 8 | Solomon Islands | 2696.64 | 70.17 | 1.93 | 0.28 |
| 2019 | 9 | Solomon Islands | 2727.90 | 70.38 | 1.55 | 0.27 |
| 2020 | 10 | Solomon Islands | 2607.48 | 70.20 | 1.45 | 0.27 |
|  |  |  |  |  |  |  |
| 1999 | -10 | Vietnam | 2363.73 | 72.32 | 1.76 | - |
| 2000 | -9 | Vietnam | 2552.68 | 72.46 | 3.17 | 0.41 |
| 2001 | -8 | Vietnam | 2743.48 | 72.65 | 3.25 | 0.42 |
| 2002 | -7 | Vietnam | 2932.06 | 72.80 | 3.43 | 0.42 |
| 2003 | -6 | Vietnam | 3163.51 | 72.98 | 3.83 | 0.43 |
| 2004 | -5 | Vietnam | 3457.79 | 73.14 | 4.51 | 0.45 |
| 2005 | -4 | Vietnam | 3797.05 | 73.27 | 4.71 | 0.46 |
| 2006 | -3 | Vietnam | 4146.95 | 73.32 | 5.21 | 0.47 |
| 2007 | -2 | Vietnam | 4519.05 | 73.44 | 5.69 | 0.48 |
| 2008 | -1 | Vietnam | 4819.01 | 73.41 | 6.28 | 0.49 |
| **2009** | **0** | **Vietnam** | **5059.34** | **73.50** | **6.76** | **0.50** |
| 2010 | 1 | Vietnam | 5391.17 | 73.51 | 7.44 | 0.51 |
| 2011 | 2 | Vietnam | 5793.93 | 73.69 | 7.89 | 0.52 |
| 2012 | 3 | Vietnam | 6364.98 | 73.70 | 8.09 | 0.53 |
| 2013 | 4 | Vietnam | 6724.64 | 73.78 | 8.29 | 0.53 |
| 2014 | 5 | Vietnam | 7240.74 | 73.86 | 8.45 | 0.54 |
| 2015 | 6 | Vietnam | 7595.69 | 73.88 | 8.86 | 0.55 |
| 2016 | 7 | Vietnam | 8277.68 | 73.94 | 9.25 | 0.55 |
| 2017 | 8 | Vietnam | 9050.69 | 73.96 | 9.39 | 0.56 |
| 2018 | 9 | Vietnam | 9867.67 | 73.98 | 9.29 | 0.56 |
| 2019 | 10 | Vietnam | 10686.80 | 74.09 | 9.34 | 0.56 |

GDP PPP: gross domestic product at purchasing power parity; APC: total alcohol per capita consumption; year of transition from low to lower-middle income is bolded; PDR: People's Democratic Republic

## S2: Specification of SEM model, sensitivity analysis and cross-correlation structure

### Specification for main analysis

1. Life Expectancy Equation:

${Life Expectancy}_{t}=a_{1}{Life Expectancy}_{t-1}+ \beta_{1}\mathrm{GDP}_{t}+\beta_{2}{APC}_{t}+ Ɛ_{1t}$ (1)

where
 - Life Expectancy at time t depends on:
 - Its value at the previous time step (${Life Expectancy}_{t-1}$).
 - GDP, APC, at time t.
 - A random error term ($Ɛ_{1t}$).

2. APC Equation:

$\mathrm{APC}_{t}=a_{2}{APC}_{t-1}+ \Upsilon_{1}\mathrm{GDP}_{t}+ Ɛ_{2t}$ (2)

where
 - APC at time t depends on:
 - Its value at the previous time step (${APC}_{t-1}$)
 - GDP at time t.
 - A random error term ($Ɛ_{2t}$)

### Specification for sensitivity analysis

1. Life Expectancy Equation:

${Life Expectancy}_{t}=a_{1}{Life Expectancy}_{t-1}+ \beta_{1}\mathrm{GDP}_{t}+\beta_{2}{APC}_{t}+ \beta_{3}\mathrm{Year}_{t}+ Ɛ_{1t}$ (3)
 - Life Expectancy at time t depends on:
 - Its value at the previous time step (${Life Expectancy}_{t-1}$)
 - GDP, APC, and Year at time t.
 - A random error term ($Ɛ_{1t}$).

2. APC Equation:$\mathrm{APC}_{t}=a_{2}{APC}_{t-1}+ \Upsilon_{1}\mathrm{GDP}_{t}+\Upsilon_{2}\mathrm{Year}_{t}+ Ɛ_{2t}$ (4)
 - APC at time t depends on:
 - Its value at the previous time step (${APC}_{t-1}$).
 - GDP and Year at time t.
 - A random error term ($Ɛ_{2t}$).

### Cross-correlation structure: no lag was identified

| 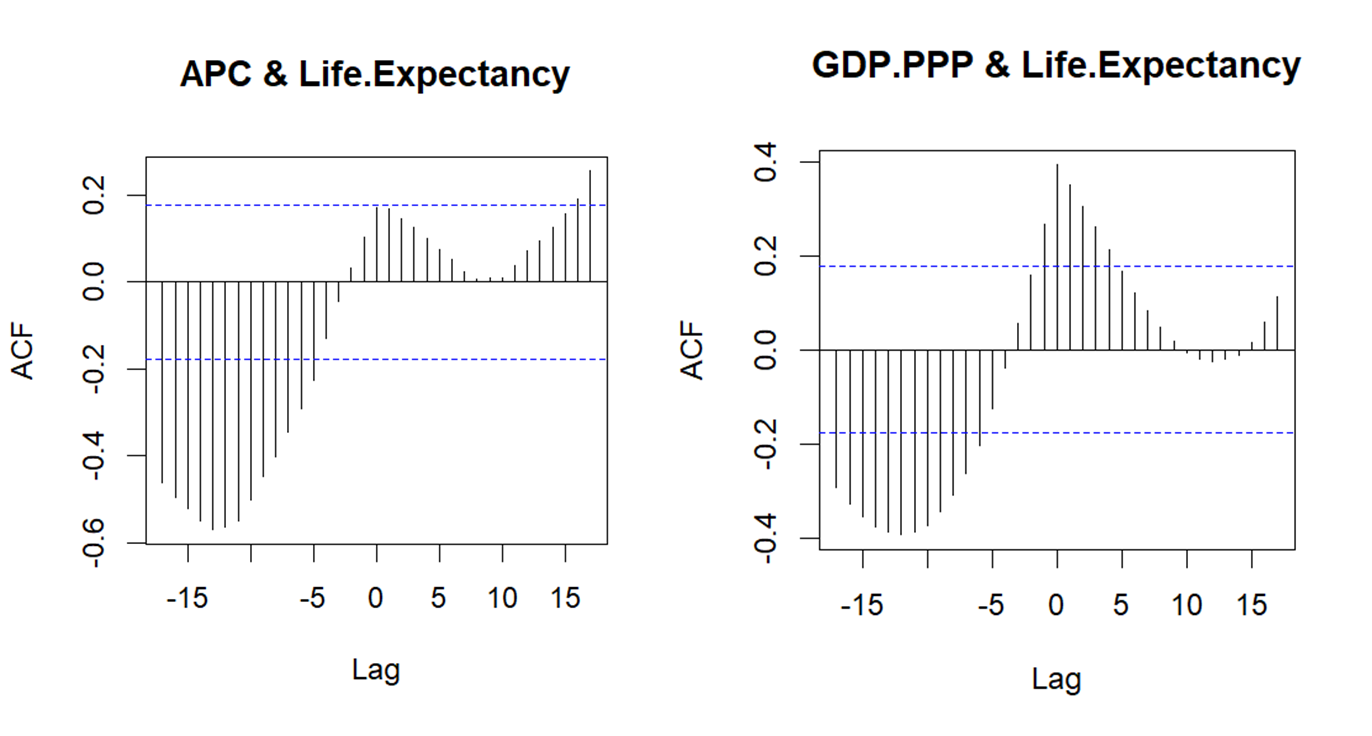 |
| --- |

## S3: Country-specific correlations and their 95% confidence intervals

### **Table 3.1:** Correlation analysis of adult alcohol per capita (in litres) consumption and life expectancy for overall data as well as before and after the transition from low- to lower-middle income

| **Country** |  | **Adult alcohol per capita (in litres) consumption and life expectancy** | | |
| --- | --- | --- | --- | --- |
|  |  | **Before** | **After** | **Overall** |
| **Cambodia** | *Total* | 0.95 (0.82, 0.99)*** | 0.71 (-0.24, 0.97) | 0.95 (0.85, 0.98)*** |
|  | *Male* | 0.95 (0.83, 0.99)*** | 0.73 (-0.20, 0.97) | 0.95 (0.86, 0.98)*** |
|  | *Female* | 0.95 (0.81, 0.99)*** | 0.59 (-0.43, 0.95) | 0.94 (0.84, 0.98)*** |
|  |  |  |  |  |
| **Lao PDR** | *Total* | 0.62 (0.03, 0.89)* | 0.60 (0.01, 0.88)* | 0.87 (0.71, 0.95)*** |
|  | *Male* | 0.65 (0.08, 0.90)* | 0.59 (-0.02, 0.88) | 0.88 (0.72, 0.95)*** |
|  | *Female* | 0.58 (-0.03, 0.88) | 0.62 (0.04, 0.89)* | 0.87 (0.69, 0.94)*** |
|  |  |  |  |  |
| **Mongolia** | *Total* | 0.59 (-0.01, 0.88) | 0.07 (-0.55, 0.64) | 0.81 (0.59, 0.92)*** |
|  | *Male* | 0.70 (0.16, 0.91)* | 0.06 (-0.56, 0.64) | 0.81 (0.59, 0.92)*** |
|  | *Female* | 0.50 (-0.14, 0.85) | 0.08 (-0.54, 0.65) | 0.78 (0.52, 0.91)*** |
|  |  |  |  |  |
| **Papua New Guinea** | *Total* | -0.62 (-0.89, -0.04)* | 0.21 (-0.45, 0.72) | -0.57 (-0.81, -0.19)** |
|  | *Male* | -0.63 (-0.89, -0.04)* | 0.20 (-0.45, 0.72) | -0.60 (-0.82, -0.22)** |
|  | *Female* | -0.59 (-0.88, 0.02) | 0.20 (-0.45, 0.72) | -0.55 (-0.80, -0.16)** |
|  |  |  |  |  |
| **Solomon** | *Total* | 0.60 (0.01, 0.88)* | 0.82 (0.42, 0.95)** | 0.71 (0.40, 0.87)** |
|  | *Male* | 0.62 (0.03, 0.89)* | 0.81 (0.40, 0.95)** | 0.71 (0.40, 0.87)** |
|  | *Female* | 0.54 (-0.09, 0.86) | 0.83 (0.47, 0.96)** | 0.72 (0.42, 0.88)** |
|  |  |  |  |  |
| **Vietnam** | *Total* | 0.95 (0.83, 0.99)*** | 0.96 (0.87, 0.99)*** | 0.98 (0.94, 0.99)*** |
|  | *Male* | 0.96 (0.83, 0.99)*** | 0.96 (0.84, 0.99)*** | 0.98 (0.95, 0.99)*** |
|  | *Female* | 0.95 (0.82, 0.99)*** | 0.96 (0.84, 0.99)*** | 0.97 (0.92, 0.99)*** |

*** p < .0001; ** p < .01; * p < .05

PDR: People's Democratic Republic

### **Table 3.2**: Correlation analysis of GDP PPP and adult alcohol per capita (in litres) consumption for overall data as well as prior to and following the transition from low- to lower-middle income

| **Country** | **Adult alcohol per capita (in litres) consumption and GDP PPP** | | |
| --- | --- | --- | --- |
|  | **Before** | **After** | **Overall** |
| **Cambodia** | 0.96 (0.86, 0.99)*** | 0.38 (-0.62, 0.91) | 0.93 (0.81, 0.98)*** |
| **Lao PDR** | 0.69 (0.15, 0.91)* | 0.63 (0.04, 0.89)* | 0.84 (0.63, 0.93)*** |
| **Mongolia** | 0.81 (0.41, 0.95)** | 0.21 (-0.45, 0.72) | 0.85 (0.66, 0.94)*** |
| **Papua New Guinea** | -0.27 (-0.75, 0.40) | 0.27 (-0.39, 0.75) | -0.47 (-0.75, -0.05)* |
| **Solomon** | 0.66 (0.09, 0.90)* | 0.75 (0.28, 0.93)** | 0.66 (0.32, 0.85)** |
| **Vietnam** | 0.98 (0.93, 1.00)*** | 0.92 (0.71, 0.98)*** | 0.94 (0.85, 0.97)*** |

*** p < .0001; ** p < .01; * p < .05

GDP PPP: gross domestic product at purchasing power parity; PDR: People's Democratic Republic

## S4: Sensitivity analysis of the SEM model: relationship between economic development, level of alcohol consumption, and life expectancy (GDP PPP in units of $ 1000 Int.)

| Response | Predictor | Estimate | Std.Error | DF | Crit.Value | P.Value |
| --- | --- | --- | --- | --- | --- | --- |
| Life Expectancy | GDP PPP | 0.0051 | 0.0020 | 113 | 2.59 | 0.01 |
| Life Expectancy | APC | 0.0003 | 0.0014 | 113 | 0.22 | 0.82 |
| Life Expectancy | Year | 0.0031 | 0.0005 | 113 | 6.56 | 0 |
| Life Expectancy | Country | - | - | 5 | 229.68 | 0 |
| Life Expectancy | Country = Lao People's Democratic Republic (2010) | 4.1496 | 0.007 | 113 | 590.46 | 0 |
| Life Expectancy | Country = Papua New Guinea (2008) | 4.1533 | 0.0048 | 113 | 858.05 | 0 |
| Life Expectancy | Country = Mongolia (2007) | 4.18 | 0.0064 | 113 | 652.66 | 0 |
| Life Expectancy | Country = Cambodia (2015) | 4.2207 | 0.0055 | 113 | 764.89 | 0 |
| Life Expectancy | Country = Solomon Islands (2010) | 4.2404 | 0.0057 | 113 | 745.90 | 0 |
| Life Expectancy | Country = Vietnam (2009) | 4.29 | 0.0041 | 113 | 1041.06 | 0 |
| APC | GDP PPP | 0.8640 | 0.0528 | 115 | 16.38 | 0 |
| APC | Country | - | - | 5 | 113.63 | 0 |
| APC | Country = Solomon Islands (2010) | 2.9123 | 0.2635 | 115 | 11.05 | 0 |
| APC | Country = Papua New Guinea (2008) | 3.0272 | 0.2497 | 115 | 12.12 | 0 |
| APC | Country = Mongolia (2007) | 4.0614 | 0.2787 | 115 | 14.57 | 0 |
| APC | Country = Vietnam (2009) | 5.2568 | 0.2493 | 115 | 21.09 | 0 |
| APC | Country = Cambodia (2015) | 6.789 | 0.271 | 115 | 25.05 | 0 |
| APC | Country = Lao People's Democratic Republic (2010) | 9.638 | 0.2393 | 115 | 40.28 | 0 |

## **S5: Estimated loss of life expectancy due to alcohol consumption (direct estimation)**

**Table 1**: Life expectancies and the estimated loss of life expectancy due to alcohol consumption

| **Country** |  | **Life Expectancy** | **Adjusted Life Expectancy** | **Estimated Loss of Life Expectancy** |
| --- | --- | --- | --- | --- |
| **Cambodia** | *Male* | 66.20 | 68.23 | 2.03 |
|  | *Female* | 71.89 | 72.61 | 0.72 |
| **Lao People's Democratic Republic** | *Male* | 62.44 | 64.85 | 2.42 |
|  | *Female* | 67.38 | 68.11 | 0.73 |
| **Mongolia** | *Male* | 58.77 | 61.80 | 3.03 |
|  | *Female* | 67.12 | 68.50 | 1.38 |
| **Papua New Guinea** | *Male* | 61.52 | 62.33 | 0.81 |
|  | *Female* | 65.40 | 65.61 | 0.21 |
| **Solomon Islands** | *Male* | 61.30 | 62.02 | 0.72 |
|  | *Female* | 66.40 | 66.61 | 0.21 |
| **Vietnam** | *Male* | 68.42 | 69.98 | 1.56 |
|  | *Female* | 77.15 | 77.46 | 0.31 |
| **Mean** | ***Male*** *[95% CI]* | **63.11**  [59.38 to 66.82] | **64.87**  [61.19 to 68.55] | **1.76**  [0.81 to 2.72] |
|  | ***Female*** *[95% CI]* | **69.22**  [64.52 to 73.92] | **69.82**  [65.15 to 74.48] | **0.59**  [0.12 to 1.07] |
